# Vitamin D deficiency contributes to vascular damage in sustained ischemic acute kidney injury

**DOI:** 10.14814/phy2.12829

**Published:** 2016-07-01

**Authors:** Ana C. de Bragança, Rildo A. Volpini, Purvi Mehrotra, Lúcia Andrade, David P. Basile

**Affiliations:** ^1^ Division of NephrologyLaboratory of Basic Science LIM‐12University of São Paulo School of MedicineSão PauloBrazil; ^2^ Department of Cellular and Integrative PhysiologyIndiana University School of MedicineIndianapolisIndiana

**Keywords:** Acute kidney injury, CD4^+^ lymphocytes, CD8^+^ lymphocytes, cytokines, ischemia reperfusion injury, renal microvascular density, T‐reg cells, vitamin D deficiency

## Abstract

Reductions in renal microvasculature density and increased lymphocyte activity may play critical roles in the progression of chronic kidney disease (CKD) following acute kidney injury (AKI) induced by ischemia/reperfusion injury (IRI). Vitamin D deficiency is associated with tubulointerstitial damage and fibrosis progression following IRI‐AKI. We evaluated the effect of vitamin D deficiency in sustained IRI‐AKI, hypothesizing that such deficiency contributes to the early reduction in renal capillary density or alters the lymphocyte response to IRI. Wistar rats were fed vitamin D‐free or standard diets for 35 days. On day 28, rats were randomized into four groups: control, vitamin D deficient (VDD), bilateral IRI, and VDD+IRI. Indices of renal injury and recovery were evaluated for up to 7 days following the surgical procedures. VDD rats showed reduced capillary density (by cablin staining), even in the absence of renal I/R. In comparison with VDD and IRI rats, VDD+IRI rats manifested a significant exacerbation of capillary rarefaction as well as higher urinary volume, kidney weight/body weight ratio, tissue injury scores, fibroblast‐specific protein‐1, and alpha‐smooth muscle actin. VDD+IRI rats also had higher numbers of infiltrating activated CD4^+^ and CD8^+^ cells staining for interferon gamma and interleukin‐17, with a significant elevation in the Th17/T‐regulatory cell ratio. These data suggest that vitamin D deficiency impairs renal repair responses to I/R injury, exacerbates changes in renal capillary density, as well as promoting fibrosis and inflammation, which may contribute to the transition from AKI to CKD.

## Introduction

Mortality rates in patients with acute kidney injury (AKI) remain unacceptably high (Basile et al. 2001; de Araujo et al. 2005; Wu et al. 2014). In addition, several studies have suggested that AKI can be a risk factor for the development of chronic kidney disease (CKD). Animal models that mimic AKI, such as the ischemia/reperfusion injury (IRI) model, have long been used to study the injury process, as well as subsequent processes of repair and recovery (Basile et al. 2011; Goncalves et al. 2014). The pathophysiology of renal IRI appears to involve a complex exchange between renal hemodynamics, tubular injury, and the inflammatory process, activating pathways of proliferation and cell death (Megyesi et al. 1996; Moe 2008).

The renal vasculature is fundamentally involved in the response to IRI (Basile 2007), which alters its structure and function. In contrast with the cells of the proximal tubule, those of the renal vasculature have a poor capacity for repair, leading to a persistent 30–50% reduction in vascular density following IRI. The reduction in vascular density is thought to promote hypoxia and impair hemodynamic responses for sodium handling, as well as potentially predisposing rats to CKD and hypertension (Basile et al. 2011).

Although it has long been known that mortality in CKD is related to cardiovascular diseases and infections (Goncalves et al. 2014), recent studies have suggested that vitamin D deficiency is also involved (Patel and Singh 2009; Goncalves et al. 2014). Vitamin D is a hormone precursor that is involved in the regulation of several key physiological functions, such as osteogenesis and the maintenance of mineral homeostasis. It is well known that vitamin D synthesis begins in the skin, and that the metabolism of vitamin D continues in the liver and the kidney to produce the biologically active form, 1,25‐dihydroxyvitamin D3 [1,25 (OH)_2_D_3_] or calcitriol (Lanske and Razzaque 2007). In addition, to perform its functions, calcitriol must bind to the vitamin D receptor (VDR), which is its cognate nuclear receptor (Dusso et al. 2005). The VDR is present in the vasculature, and normal vitamin D status may protect against endothelial dysfunction and vascular diseases, including atherosclerosis (Kienreich et al. 2013). In vitro studies have reported that vitamin D improves the angiogenic properties of endothelial progenitor cells, probably through an increase in vascular endothelial growth factor (VEGF) expression. Because vitamin D deficiency is associated with impairment of endothelial function, it can reduce the number of endothelial progenitor cells (Dusso 2012). Some studies have shown that low levels of vitamin D are associated with endothelial dysfunction (Kienreich et al. 2013), whereas others have demonstrated that vitamin D can increase nitric oxide production, decrease expression of adhesion molecules in endothelial cells, or reduce macrophage infiltration (Molinari et al. 2011; Kienreich et al. 2013). Recently, we studied the impact of vitamin D deficiency in renal IRI (de Braganca et al. 2015). In that study, we found that, compared with IRI rats on a standard diet, rats in the vitamin D deficient (VDD)‐IRI group presented a more severe decrease in glomerular filtration rate, greater urinary protein excretion, a higher kidney weight/body weight (KW/BW) ratio, and lower renal aquaporin 2 expression, as well as greater morphological damage, characterized by increased interstitial area and tubular necrosis. Our results suggested that the severity of tubular damage in IRI might be associated with downregulation of VDRs and p21. However, in light of the aforementioned evidence that low levels of vitamin D are associated with endothelial dysfunction, this study was conducted to test the hypothesis that vitamin D deficiency also impairs renal vascular density in response to IRI, providing a potential mechanism by which the transition from AKI to CKD may be exacerbated in vitamin D deficiency.

## Materials and Methods

### Animals

Animal procedures were conducted in accordance with the policies of the National Institutes of Health (NIH) *Guide for the Care and Use of Laboratory Animals*. All protocols had received prior approval by the Institutional Animal Care and Use Committee at the Indiana University School of Medicine.

Male Wistar rats (180–200 g) were housed in pairs in standard shoe‐box cages and exposed to a 12/12‐h light/dark cycle. During the 35‐day experiment, rats received a vitamin D‐free diet or a standard diet and were given free access to tap water. Rats were divided into four groups: control (receiving a standard diet and subjected to sham surgery; *n *=* *6); VDD (receiving a vitamin D‐free diet and subjected to sham surgery; *n *=* *5); IRI (receiving a standard diet and subjected to bilateral renal ischemia for 45 min on day 28; *n *=* *7); and VDD+IRI (receiving a vitamin D‐free diet and subjected to bilateral renal ischemia for 45 min on day 28; *n *=* *7).

### Diet

The animals received either a vitamin D‐free diet (catalog number 960074; MP Biomedicals, Irvine, CA) or a standard vitamin D (control) diet (catalog number 960226; MP Biomedicals). The composition of the standard diet is as follows: vitamin‐free casein (13.0%); whole wheat flour (75.0%); corn oil (3.0%); alphacel, nonnutritive bulk (5.0%); calcium carbonate (0.8%); vitamin D (5 IU/g of diet); and calcium phosphorous‐free salt mixture (2.0%). The vitamin D‐free diet has the same composition as the standard diet except that it does not contain vitamin D. We have previously shown that receiving a vitamin D‐free diet for 30 and 90 days can decrease the serum levels of vitamin D (Goncalves et al. 2014; de Braganca et al. 2015).

### Ischemia/reperfusion

On day 28, rats were anesthetized with ketamine (100 mg/kg ip) and pentobarbital sodium (25 mg/kg ip), after which they were placed on a heated surgical table. After a midline incision had been made, microvascular clamps were placed on the renal pedicles of both kidneys. After 45 min, the clamps were released and reperfusion was visualized. Sham‐operated groups were exposed to the same treatments except the kidneys were not touched.

### Measurement of renal function

Parameters of renal function were measured at 48 h and on day 7 post‐surgery. Tail blood samples were collected under light isoflurane anesthesia into heparinized tubes, and plasma was obtained after centrifugation. To measure water intake and urine output, we collected urine for 24 h in metabolic cages. Serum and urine creatinine were determined using a creatinine analyzer (Pointe 180 QT; Pointe Scientific, Canton, MI), and creatinine clearance was determined.

At the end of the study (day 7 post‐surgery), rats were deeply anesthetized with ketamine (50–100 mg/kg ip) and pentobarbital sodium (25 mg/kg ip). Right kidneys were removed and weighed to obtain the kidney weight to body weight ratio (KW/BW ratio). Kidneys were harvested and frozen or fixed for immunohistochemical and flow cytometry procedures as described in detail below.

### Light microscopy

Histological sections (4 *μ*m) of kidney tissue were stained with periodic acid–Schiff and examined under light microscopy. For histomorphometry, the images obtained by microscopy were captured on video via an image analyzer (AxioVision; Carl Zeiss, Eching, Germany). In 40–60 grid fields (0.245 mm^2^ each; magnification, ×400), we graded the proportional renal damage (tubular epithelial swelling, vacuolar degeneration, necrosis, and desquamation): 0, <5%; I, 5–25%; II, 26–50%; III, 51–75%; and IV, >75%. To minimize bias in the morphometric analysis, the observer was blinded to the treatment groups. The mean scores were calculated by rat and by group (Miyaji et al. 2001).

### Immunohistochemistry

We used the following antibodies: rabbit anti‐cablin antibody, as described previously (Charron et al. 1999); rabbit polyclonal antibody to fibroblast‐specific protein 1, also known as S100A4 (FSP1/S100A4, 1:300 for 60 min at 20°C; Dako, Glostrup, Denmark); rabbit monoclonal antibody to alpha‐smooth muscle actin (*α*‐SMA, 1:500 overnight at 4°C; Millipore, Billerica, MA); and mouse monoclonal antibody to VEGF (1:100 overnight at 4°C; Santa Cruz Biotechnology, Santa Cruz, CA). We subjected 4‐*μ*m kidney tissue sections to immunohistochemical reaction according to the protocol for each primary antibody. Reaction products were detected by avidin‐biotin‐peroxidase (Vector Laboratories, Burlingame, CA). The color reaction was developed in 3,3‐diaminobenzidine (Sigma, St. Louis, MO) and hydrogen peroxide. Counterstaining was with Harris’ hematoxylin. Negative controls for FSP1/S100A4, *α*‐SMA, and VEGF consisted of replacement of the primary antibody with normal rabbit IgG for polyclonal antibodies and with mouse IgG for monoclonal antibodies, at equivalent concentrations (supplemental data).

We analyzed 30–50 fields, each for the renal cortex and renal medulla. For FSP1/S100A4, the results of immunoreactions were quantified by counting the number of positive cells per 0.087‐mm^2^ field and averaging the number of cells per field for each section. To evaluate immunoreactivity to *α*‐SMA and VEGF, the volume ratios of positive areas of renal tissue (in %) (Lancas et al. 2011), determined by the color limit, were obtained by image analysis with the program Image‐Pro Plus, version 4.1 (Media Cybernetics, Silver Spring, MD) on a computer coupled to a microscope (Axioskop 40; Carl Zeiss) and a digital camera.

To evaluate renal blood vessels, immunofluorescent staining with the endothelial‐specific marker cablin was carried out as in previous studies (Basile et al. 2011), using tissues that were immersion fixed in 100% ice‐cold methanol and sectioned at ≈100 *μ*m with a vibratome. First, endogenous peroxidases in free‐floating sections were inhibited by treatment with 2.1% H_2_O_2_ for 45 min followed by a 30‐min incubation in blocking solution consisting of 0.1 mol/L Tris, pH 7.5, containing 0.9% NaCl, 0.5% bovine serum albumin (USB Corporation, Cleveland, OH), 0.05% fish skin gelatin (Sigma), and 0.1% Triton X‐100 (Sigma). Rabbit anti‐cablin (a gift from Dr. Robert Bacallao) was incubated for ≈18 h at 4°C with gentle agitation at a concentration of 1:200,000 dilution or 0.0624 *μ*g/mL, respectively. The tissues were rinsed extensively for up to 3 h at room temperature in blocking solution with at least three changes. Tissues were then incubated for up to ≈18 h in goat anti‐rabbit‐horseradish peroxidase (1:10,000, 4030‐05; Southern Biotech, Birmingham, AL). Following rinsing, the signal was developed with a tyramide Cy3 amplification kit (NEL744001KT; Perkin‐Elmer, Waltham, MA), according to the manufacturer's instructions. After a final rinse, the tissues were mounted on slides and coverslipped in a solution of 50% glycerol/20% Mowiol (Calbiochem, San Diego, CA), after which they were allowed to dry overnight. For fluorescence detection, images were obtained using a confocal microscope (LSM NLO; Carl Zeiss) equipped with Ar and HeNe lasers. For fluorescein, Cy3 was induced by excitation at 545 nm and detected at 565–615 nm. For the quantification of vascular density, a minimum of five random images from the cortex and outer medulla of each kidney were analyzed using Image J software; images were overlaid with a 12 × 12 grid; and vessel density was determined from the number of positively stained vessels that intersected the overlaid gridlines. Data were normalized and expressed as the mean percent change relative to those obtained for control rats.

### Flow cytometry

Harvested kidneys were minced and digested in liberase (2 *μ*g/mL; Roche, Basel, Switzerland) for 15 min at 37°C with the gentleMACs dissociator (Miltenyi Biotec, Bergisch Gladbach, Germany). The digested tissue was filtered through a 100‐*μ*m filter mesh and washed with tissue culture medium. The lymphocytes were separated by Percoll (Sigma) and counted with a hemocytometer. To evaluate T lymphocytes, the cells were stained with the following antibodies: phycoerythrin (PE)‐Cy7‐conjugated rat CD4; Alexa 647‐conjugated anti‐rat CD8a; fluorescein isothiocyanate (FITC)‐conjugated anti‐rat CD25; and PE‐conjugated anti‐rat CD62L. To evaluate the cytokines secreted by the T cells, the cells were stained for CD4 surface marker, permeabilized with saponin, and stained with the antibodies FITC‐conjugated anti‐rat interferon gamma (IFN‐*γ*), PE‐conjugated anti‐rat interleukin (IL)‐4, FITC‐conjugated anti‐rat IL‐17, and PE‐conjugated anti‐rat tumor necrosis factor‐alpha. B cells were stained with FITC‐conjugated antibody against RTIB, and macrophages were stained with PE‐conjugated anti‐CD11b/c antibody. All antibodies were obtained from BD Biosciences (San Jose, CA). Cells were scanned using flow cytometry (FACSCalibur, BD Biosciences), and scans were analyzed using Flowjo software (Tree Star, Ashland, OR). The lymphocyte gating strategy was used exactly as outlined and illustrated previously (Mehrotra et al. 2015). The data were expressed as the total number of specific cells per gram of kidney.

### Statistical analysis

All quantitative data were expressed as mean ± SEM. We analyzed differences among the means of multiple parameters by one‐way analysis of variance followed by the Student‐Newman‐Keuls test. Values of *P *<* *0.05 were considered statistically significant.

## Results

### Renal function

As expected, at 48 h and at 7 days after the IRI procedure, IRI and VDD+IRI rats showed higher plasma levels of creatinine and lower creatinine clearance compared with control and VDD rats (Table 1). In addition, VDD+IRI rats displayed higher urine output and a tendency toward increased water intake compared with control, VDD, and IRI rats (Table 1). Furthermore, the KW/BW ratio was increased in the IRI and VDD+IRI groups compared with the control and VDD groups. Moreover, the KW/BW ratio was higher in the VDD+IRI group when compared with the IRI group (Table 1).

**Table 1:** Renal functional parameters in rats fed a standard or vitamin D‐free diet and subjected or not to renal ischemia/reperfusion**.**

Parameter	Control (*n *=* *6)	VDD (*n *=* *5)	IRI (*n *=* *7)	VDD+IRI (*n *=* *7)
2 days post‐injury				
SCr (mg/dL)	0.45 ± 0.03	0.40 ± 0.00	1.88 ± 0.44^b^^,^ f	1.93 ± 0.37^c^^,^ f
CCr (mL/min/100 g)	0.42 ± 0.06	0.41 ± 0.01	0.19 ± 0.03^b^^,^ e	0.16 ± 0.04^b^^,^ e
7 days post‐injury				
SCr (mg/dL)	0.41 ± 0.03	0.48 ± 0.03	0.55 ± 0.03^c^	0.55 ± 0.02^c^
CCr (mL/min/100 g BW)	0.46 ± 0.03	0.50 ± 0.04	0.37 ± 0.01^f^	0.37 ± 0.01^f^
Water intake (mL/day)	26.20 ± 2.31	30.50 ± 4.83	37.43 ± 3.20	44.0 ± 9.8
Urine output (mL/day)	10.50 ± 0.84	17.40 ± 3.80	23.57 ± 2.68	27.57 ± 6.31^c^
KW/BW ratio	0.34 ± 0.009	0.33 ± 0.005	0.50 ± 0.031^a^^,^ d	0.58 ± 0.030^a^^,^ d ^,^ i

Control, sham‐operated (standard diet); VDD, vitamin D deficient (vitamin D‐free diet); IRI, ischemia/reperfusion injury (standard diet and subjected to bilateral renal ischemia for 45 min on day 28); VDD+IRI, vitamin D deficient+ischemia/reperfusion injury (vitamin D‐free diet and subjected to bilateral renal ischemia for 45 min on day 28); SCr, serum creatinine; CCr, clearance creatinine; KW/BW ratio, kidney weight/body weight ratio. Data are mean ± SEM. ^a^ *P* < 0.001, ^b^ *P* < 0.01 and ^c^ *P* < 0.05 versus control; ^d^ *P* < 0.001, ^e^ *P* < 0.01 and ^f^ *P* < 0.05 versus VDD; and ^i^ *P *< 0.05 versus IRI.

### Renal vascular density

**Figure 1:**
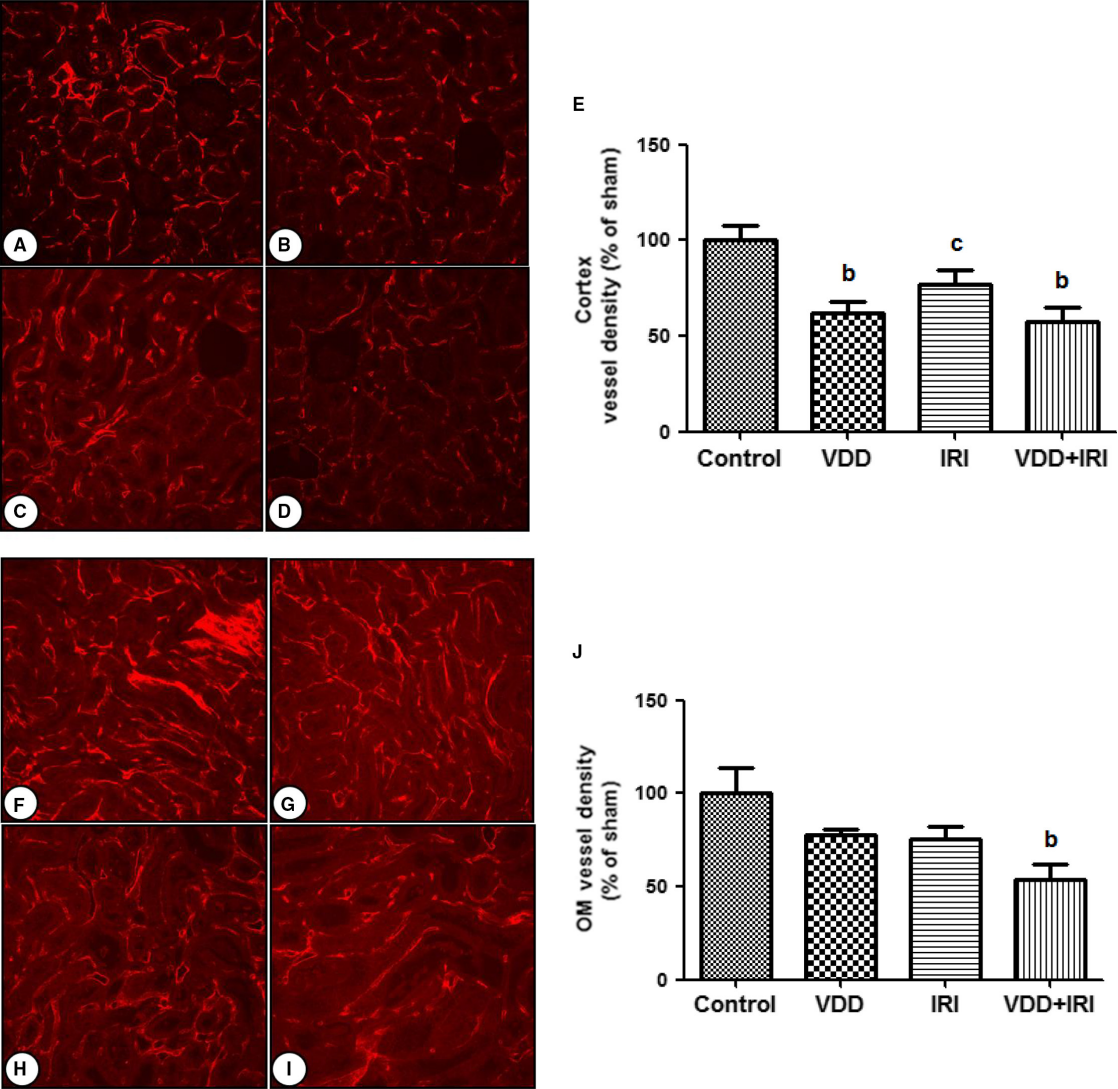
Renal vascular density on day 7 post‐IRI. Cortex tissue analysis: (A) Control; (B) VDD; (C) IRI; (D) VDD+IRI; (E) Bar graph of cortex vessel density (% of sham). Medullary tissue analysis: (F) Control; (G) VDD; (H) IRI; (I) VDD+IRI; (J) Bar graph of vessel density (% of control value) in the outer medulla. Shown are data from morphometric analysis of cablin immunofluorescence‐stained microvessel structures intersecting arbitrary grid lines and expressed as a percentage of the control group value. Data are mean ± SEM. ^b^*P* < 0.01 and ^c^*P* < 0.05 versus control.

As can be seen in Figure 1A–E, the vascular density (in %) of the renal cortex was lower in the VDD, IRI, and VDD+IRI rats than in the control rats (61.86 ± 5.91, 77.32 ± 7.14, and 57.17 ± 7.73, respectively, vs. 100.0 ± 7.60; *P *<* *0.01, *P *<* *0.05, and *P *<* *0.01, respectively). In the renal outer medulla, VDD and IRI rats both showed significant reductions in the mean percent vascular density relative to control rats (77.45 ± 3.33 and 75.50 ± 6.91, respectively, vs. 100.0 ± 14.08), whereas the VDD+IRI combination resulted in significantly lower vascular density (53.42 ± 8.58) than did either vitamin D deficiency or IRI alone (*P *<* *0.05), as shown in Figure 1F–J.

### Protein expression of FSP1/S100A4 and *α*‐SMA

**Figure 2:**
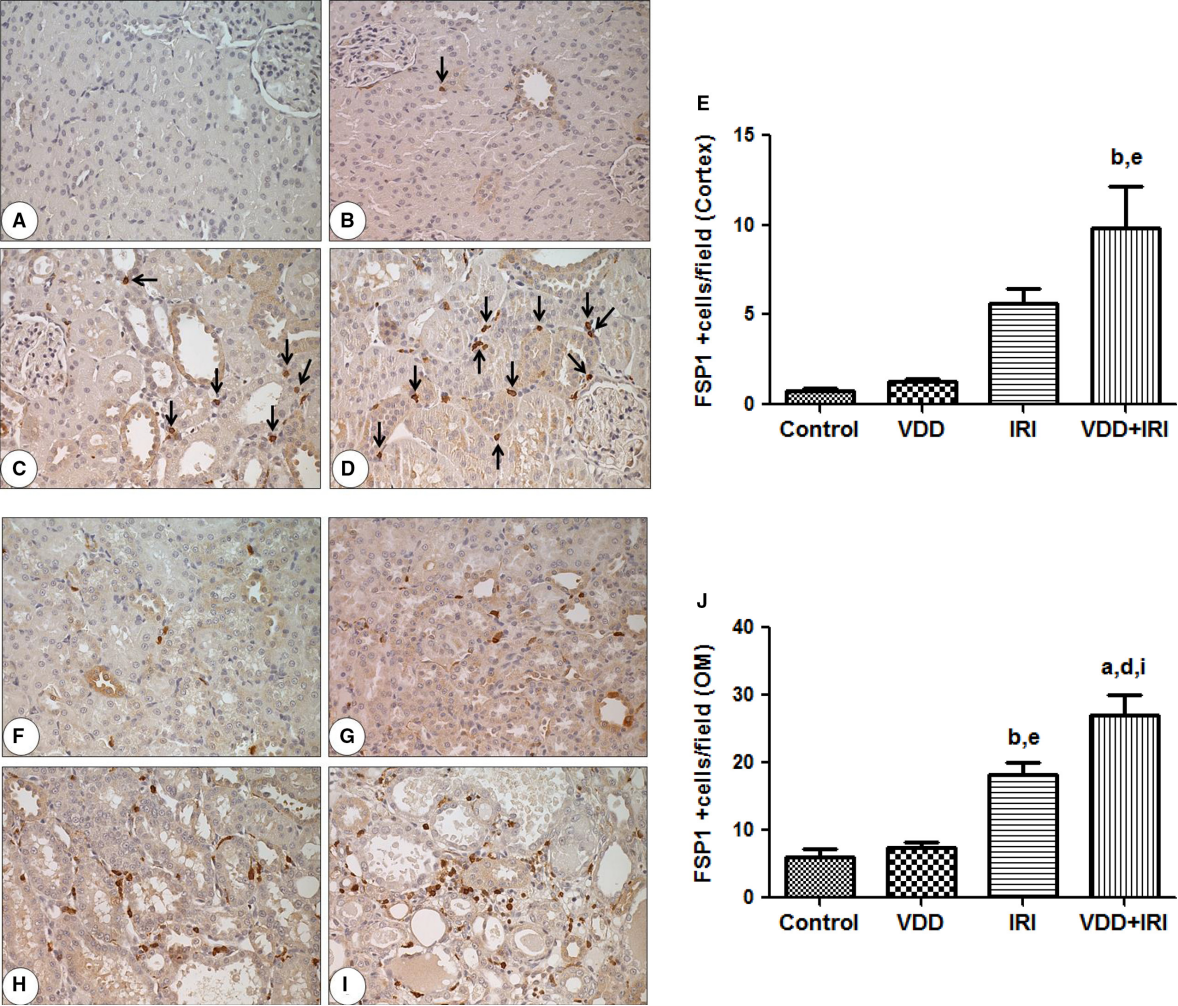
Immunohistochemical analysis of FSP1/S100A4 expression in rat kidney tissue on day 7 post‐IRI. Immunostaining (black arrow) for FSP1/S100A4 in the cortex. Cortex tissue analysis: (A) Control group; (B) VDD; (C) IRI; (D) VDD+IRI; (E) Bar graph of vessel density (% of control value) in the cortex. Medullary tissue analysis: (F) Control group; (G) VDD; (H) IRI; (I) VDD+IRI; (J) Bar graph of FSP1 expression in the outer medulla. Data are mean ± SEM. ^a^*P* < 0.001 and ^b^*P* 0.01 versus control; ^d^*P* < 0.001 and ^e^*P* < 0.01 versus VDD;^i^*P* < 0.05 versus IRI.

**Figure 3:**
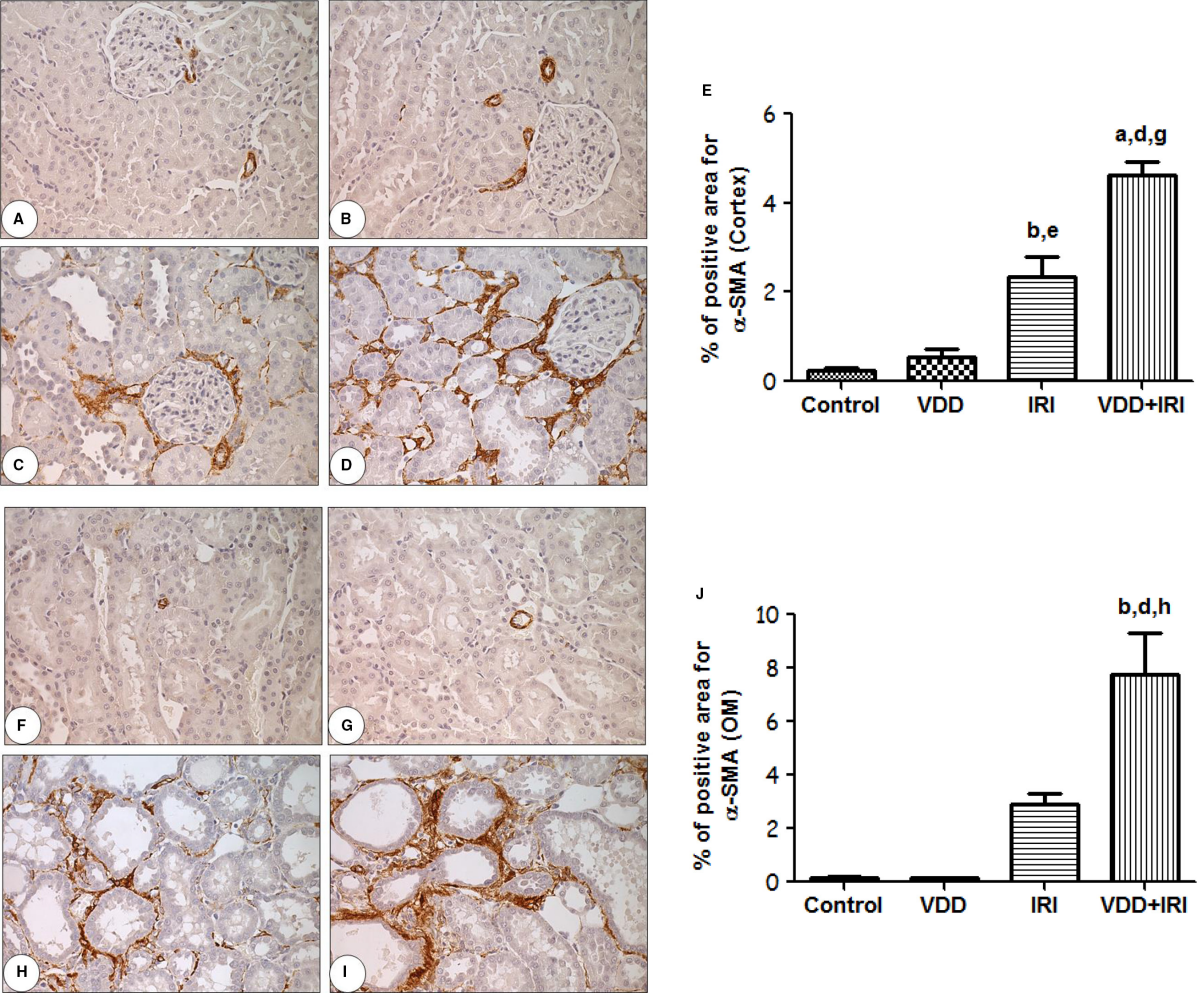
Immunohistochemical analysis of *α*‐SMA expression in rat kidney tissue on day 7 post‐IRI. Immunostaining (brown) for *α*‐SMA in kidney cortex and outer medulla. Cortex tissue analysis: (A) Control; (B) VDD; (C) IRI; (D) VDD+IRI; (E) Bar graph of vessel density (% of control value) in the cortex. Medullary tissue analysis: (F) Control group; (G) VDD; (H) IRI; (I) VDD+IRI; (J) Bar graph of *α*‐SMA expression in the outer medulla. Data are mean ± SEM. ^a^*P* < 0.001 and ^b^*P* 0.01 versus control; ^d^*P* < 0.001 and ^e^*P* < 0.01 versus VDD;^g^*P* < 0.001 and ^h^*P* < 0.01 versus IRI.

The presence of interstitial fibroblasts was assessed by immunostaining for FSP1/S100A4. Vitamin D deficiency alone did not influence the number of fibroblasts (FSP1/S100A4 + cells/0.087 mm^2^) in the renal cortex and renal medulla. IRI significantly increased the numbers of fibroblasts in the cortex and medulla, and those numbers were further increased in the VDD+IRI group in comparison with the IRI group (Fig. 2). A similar pattern of myofibroblast expression was observed when tissues were stained for *α*‐SMA. As shown in Figure 3, although the area of renal tissue staining positive for *α*‐SMA (in %) was not influenced by vitamin D deficiency alone (vs. control), that area, in the cortex and in the medulla, was significantly larger in the IRI group than in the control group. In addition, myofibroblast expression was further significantly increased in the VDD+IRI rats in comparison with the IRI rats.

### Tubular injury and inflammation

**Figure 4:**
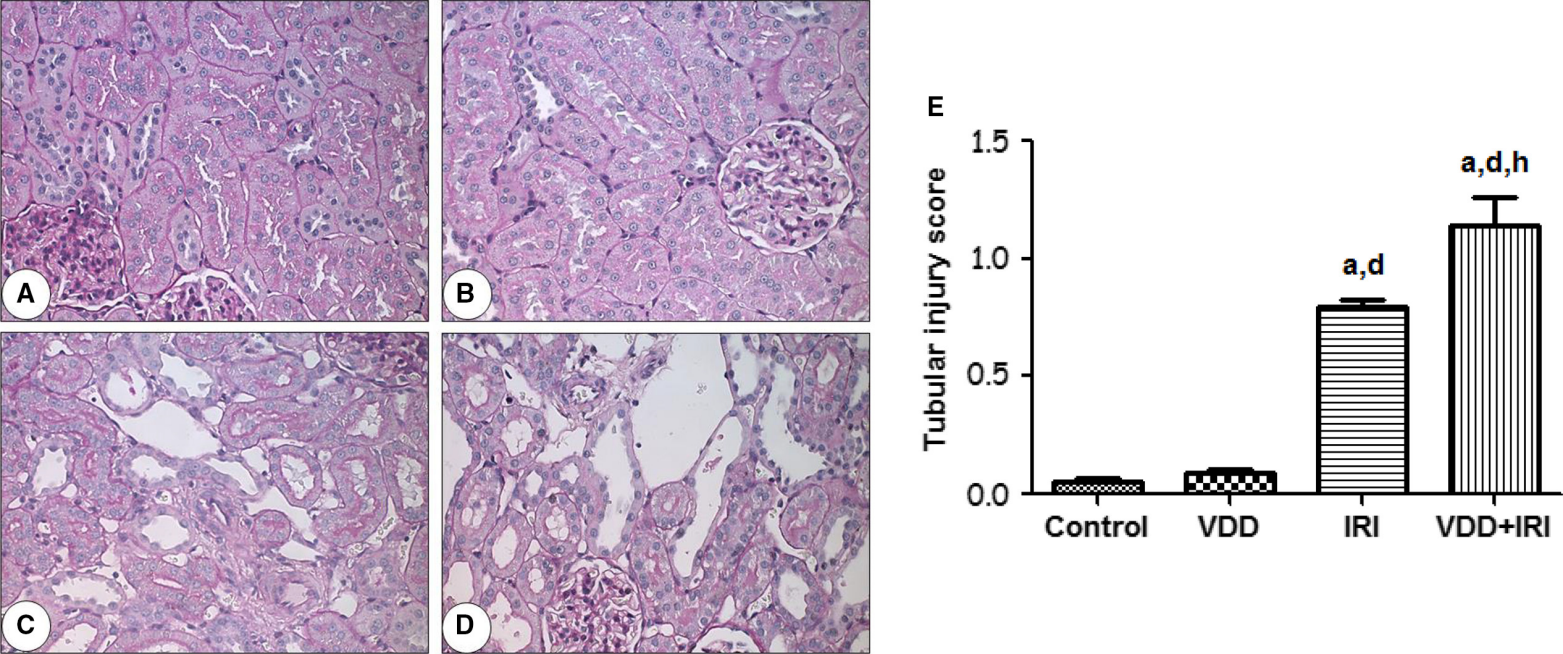
Tubular injury in rat kidney tissue on day 7 post‐IRI. Representative photomicrographs of kidney tissue samples. (A) Control; (B) VDD; (C) IRI; (D) VDD+IRI. Magnification, ×400. (E) Bar graph of tubular injury scores. Data are mean ± SEM. ^a^*P* < 0.001 versus control; ^d^*P* < 0.001 versus VDD;^h^*P* < 0.01 versus IRI.

An important feature of renal IRI is tubular repair, the success or failure of which may determine the degree to which AKI is reversed or sustained (Venkatachalam et al. 2010). As shown in Figure 4, after 7 days of recovery, there was still marked renal tubular damage in the IRI and VDD+IRI groups in comparison with the control and VDD groups (0.79 ± 0.02 and 1.14 ± 0.11 vs. 0.05 ± 0.01 and 0.08 ± 0.02, respectively; *P *<* *0.001 for all). We find it interesting that the VDD+IRI rats manifested more significant tubular damage than did the IRI rats (*P *<* *0.01), which suggests that vitamin D deficiency inhibits the process of tubular repair in response to ischemia.

Renal tissues were also prepared for analysis by flow cytometry and differential staining to investigate specific populations of T‐lymphocytes. CD4^+^ and CD8^+^ cells were low in numbers in control or VDD rats. As expected, the vitamin D‐free diet increased the number of CD4^+^ and CD8^+^ cells in postischemic rats. (Table 2). When further analyzed for specific subpopulations of T‐helper (Th) cells, the number of CD4^+^ or CD8^+^ cells stained for either IFN‐*γ* or IL‐17, markers of Th1 and Th17 cells, respectively, were undetectable in control and VDD rats. However, after 7 days of recovery from IRI, the numbers of Th1 and Th17 cells were significantly higher in the VDD+IRI group than in the IRI group (Table 2). In addition, the numbers of T‐regulatory cells, which have been considered to have a beneficial role in renal repair, were significantly lower in the VDD and IRI groups. The Th17/T‐regulatory cell ratio was increased by both vitamin D deficiency and IRI, whereas the combination of vitamin D deficiency and IRI significantly increased this ratio further in comparison with either vitamin D deficiency or IRI alone (Table 2).

**Table 2:** Flow cytometry analysis of lymphocytes from rat kidneys on day 7 post‐IRI

Parameters	Control (*n *=* *3)	VDD (*n *=* *3)	IRI (*n *=* *7)	VDD+IRI (*n *=* *7)
CD4^+^	2371 ± 457	2281 ± 1050	2175 ± 469	4432 ± 746^i^
CD8^+^	1399 ± 439	2016 ± 509	1255 ± 317	3451 ± 485^i^
CD4^+^/IL17^+^	ND	ND	424 ± 188	2055 ± 584^i^
CD4^+^/IFN‐*γ* ^+^	ND	ND	40 ± 21	237 ± 52^i^
Treg	34 ± 8.4	26 ± 2.8	12 ± 3.1	9.6 ± 2.4^i^
Th17/Treg	ND	ND	1.8 ± 0.5	27 ± 7^i^
Th1/Treg	ND	ND	20.5 ± 5.2	265.1 ± 6.9^i^

Control, sham‐operated (standard diet); VDD, vitamin D deficient (vitamin D‐free diet 35 days, sham surgery at 28 days); IRI, ischemia/reperfusion injury (standard diet 35 days subjected to bilateral renal ischemia for 45 min on day 28); VDD+IRI, vitamin D deficient+ischemia/reperfusion injury (vitamin D‐free diet 35 days and subjected to bilateral renal ischemia for 45 min on day 28); CD4^+^, CD8^+^, IFN‐*γ* ^+^, and Th17 cells defined as described previously (Mehrotra et al. 2015), T‐regulatory cells defined as % of Foxp3^+^ cells. Data are mean ± SEM. ^i^ *P* < 0.05 IRI versus VDD+IRI.

**Figure 5:**
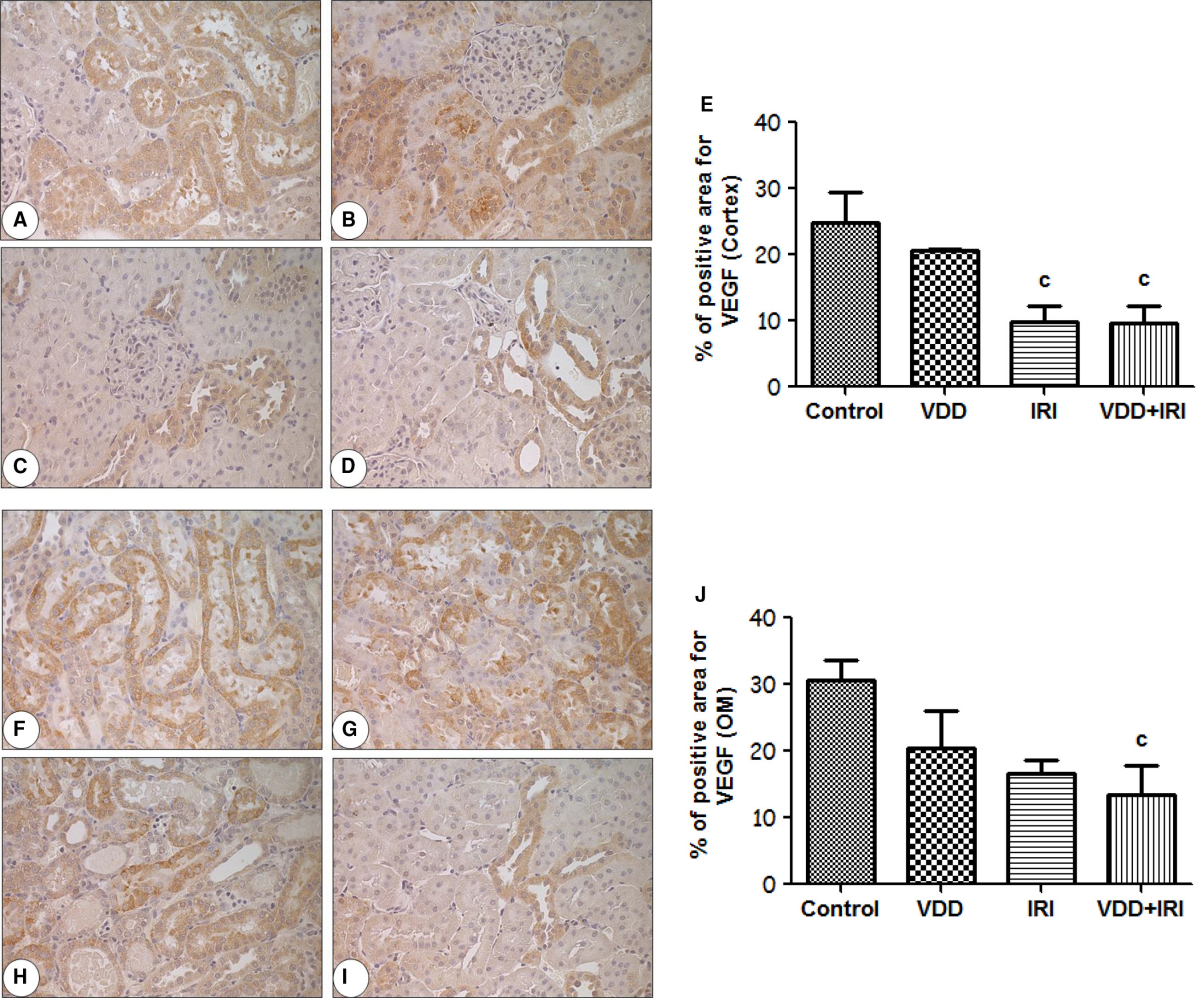
Immunohistochemical analysis of VEGF expression in rat kidney tissue on day 7 post‐IRI. Immunostaining (brown) for VEGF in the cortex and outer medulla. Cortex tissue analysis: (A) Control; (B) VDD; (C) IRI; (D) VDD+IRI; (E) Bar graph of vessel density (% of sham) in the cortex. Medullary tissue analysis: (F) Control; (G) VDD; (H) IRI; (I) VDD+IRI; (J) Bar graph of VEGF expression in the outer medulla. Data are mean ± SEM. ^c^*P* < 0.05 versus control.

To investigate potential molecular mediators by which vitamin D deficiency might reduce the efficiency of renal repair following IRI, renal cortical VEGF expression was measured by immunohistochemistry. Vitamin D deficiency tended to decrease VEGF expression (% positive tubules) relative to the control group in the cortex (Fig. 5A–E) and in the outer medulla (Fig. 5F–J), although the differences were not statistically different. Consistent with previous reports (Basile et al. 2008), renal VEGF protein expression was lower in IRI and VDD+IRI rats than in control and VDD rats, in the cortex (Fig. 5A–E) and outer medulla (Fig. 5F–J).

## Discussion

Vitamin D deficiency is present in a considerable number of critical patients and is associated with increased mortality among such patients. Clinical studies have identified low serum vitamin D as a risk factor for AKI in critically ill patients (Braun et al. 2012; Braun and Christopher 2013). In a recent study, de Boer et al. (2011) suggested that low serum vitamin D is a risk factor for progression to CKD. Various authors have suggested the potential mechanisms of how vitamin D deficiency predisposes individuals to AKI (Braun and Christopher 2013), including dysregulation of the immune system, which may predispose patients to sepsis, increasing endothelial dysfunction, and impairment of healing following renal IRI.

To investigate the effects of vitamin D deficiency, we used a rat model of vitamin D deficiency induced by removal of vitamin D from the diet for up to 35 days. Previous studies from our group have shown that this model produces an 80% reduction in circulating levels of 25(OH)D_3_ (de Braganca et al. 2015). In addition, we have shown that serum ionized calcium and phosphorus are decreased in the vitamin D deficiency model, as are glomerular filtration, urine osmolality, and the fractional excretion of calcium. We have also demonstrated an increase in urinary volume in VDD rats, as well as that vitamin D deficiency aggravates IRI‐AKI in rats (de Braganca et al. 2015). Two days after renal IRI, VDD animals presented a greater decrease in glomerular filtration rate and proteinuria compared with animals in normal diet (de Braganca et al. 2015). Notably, we demonstrated that VDD rats submitted to IRI presented lower activation of p21 protein expression, higher numbers of tubular necrotic cells, and greater macrophage infiltration (de Braganca et al. 2015). In their clinical study, de Boer et al. (2011) reported that vitamin D deficiency is a risk factor for loss of renal function and postulated that vitamin D supplementation might prevent the progression to CKD.

In this study, we have demonstrated that vitamin D deficiency in isolation impairs renal capillary density, whereas vitamin D deficiency in the setting of IRI further worsens renal capillary rarefaction, impairs tubular recovery, exacerbates fibrotic damage, and contributes to the proinflammatory milieu, all of which might promote the transition from AKI to CKD. In addition, the VDD+IRI animals presented higher urinary volume, suggesting a sustained urinary concentrating defect. In recent years, there have been many reports demonstrating a strong link between AKI and the progression to CKD (Chawla and Kimmel 2012; Coca et al. 2012). Following ischemia, the injury and repair processes begin nearly simultaneously. However, the factors that result in renal repair and differentiate between that leading to complete recovery and that leading to incomplete repair have yet to be fully elucidated. Maladaptive repair, which can be defined as a process that results in a durable reduction in renal function, usually associated with a change in renal structure, can occur in the tubular, vascular, and interstitial compartments in response to AKI, predisposing to the development of interstitial fibrosis (Basile et al. 2016). Maladaptive repair may be associated with the persistent expression of profibrogenic factors, delayed inflammation, and evidence of persistent damage (Basile et al. 2016). It is known that established CKD and AKI severity are both risk factors for chronification (Chawla et al. 2011; Belayev and Palevsky 2014), and that maladaptive repair may be more preferentially activated in both. We propose that vitamin D deficiency also shifts the balance from adaptive repair toward maladaptive repair, thus predisposing to the transition from AKI to CKD.

It is of note that, in this study, renal vascular density was lower in the VDD rats than in the control rats, even before the former had been subjected to ischemia. In addition, VDD+IRI rats presented lower vascular density in the renal outer medulla, suggesting that normalizing vitamin D status has a positive influence on renal vascular remodeling, not only in the steady state but also in response to injury. It has been shown that a reduction in capillary density after AKI can exacerbate renal hypoxia and thus promote interstitial fibrosis (Basile et al. 2001; Basile 2007; Kramann et al. 2014). For example, VEGF, an important growth factor, is produced in the kidney proximal tubule. In many models of renal disease, VEGF expression is decreased in the proximal tubule. Exogenous VEGF administered to preserve renal capillaries has a therapeutic window only in the early postinjury period (Dusso et al. 2005; Ascon et al. 2008, 2009).

The conversion of 1,25 (OH)_2_D_3_ (the active form of vitamin D) via 1‐*α*‐hydroxylase occurs in vascular smooth muscle cells and in the endothelial vascular wall. Vitamin D deficiency has been associated with endothelial dysfunction and vascular stiffness, characteristics that lead to long‐term cardiovascular morbidity and mortality. FSP1/S100A4 is widely used as a marker for the identification of fibroblasts in the renal interstitium. FSP1/S100A4 is expressed in several injured tissues including arterial smooth muscle cells. Two studies (Basile et al. 2011; Basile and Yoder 2014) suggested that there is a change in endothelial cell phenotype through the mechanism of endothelial‐to‐mesenchymal transition. The authors of those studies demonstrated that, as early as 6 h and as long as 7 days after AKI, there was colocalization of endothelial markers (CD31 and cablin) with mesenchymal markers (FSP1/S100A4), which increases the expression of fibroblasts and contributes to the loss of endothelial cells. A potential limitation of these studies, as well as of all studies identifying changes in fibroblast and myofibroblast content, is related to the specificity of the antibodies used in order to label those components. Although we utilized nonimmune IgG controls, this limitation applies to this study because the specificity of the commercially obtained antibodies was not validated with antigen adsorption control studies. Nevertheless, these antibodies have been routinely used to identify S100A4 and alpha‐smooth muscle actin in paraffin‐fixed kidney tissue (Manickam et al. 2014; Choi et al. 2015; Liang et al. 2015; Zhou et al. 2015), and the pattern of staining is consistent with fibroblasts and myofibroblasts. Therefore, we feel it is reasonable to conclude that the changes in S100A4 and SMA staining reported here likely reflect real renal injury‐induced changes in interstitial fibroblasts and myofibroblasts.

Taken together, these reports indicate that vitamin D deficiency directly impairs renal vascular endothelial function, promoting endothelial apoptosis or endothelial‐to‐mesenchymal transition and resulting in reduced vascular density. It remains unclear why vitamin D deficiency promotes greater impairment of renal capillary density, exacerbates fibrotic damage, and contributes to the proinflammatory milieu following AKI.

We also demonstrated that the number of proinflammatory lymphocytes expressing either IFN‐*γ* or IL‐17 was higher in VDD+IRI rats than in IRI rats at 7 days after IRI. In addition, there was no increase in the numbers of antiinflammatory T‐regulatory cells. In AKI, many cellular and humoral immune system components are involved in the genesis of renal injury as well as in the repair response that ensues to restore renal structure and function (Jang and Rabb 2009; Jang et al. 2009). Several studies have indicated that CD4^+^ T cells contribute directly and indirectly to the establishment of renal injury in the early phase of IRI (Rabb et al. 2000; Burne et al. 2001; Day et al. 2006), whereas CD4^−^/CD8^−^ double‐knockout mice have been found to be protected from such injury (Rabb et al. 2000). The production of IFN‐*γ* and IL‐17, characteristic of Th1 and Th17 cells, respectively, might mediate the activation of CD4^+^ T cells in AKI (Burne et al. 2001). In addition, CD8^+^ T cells isolated from postischemic kidneys produce more IFN‐*γ* than those isolated from the kidneys of normal animals (Ascon et al. 2008). T cells may also play a role in the late development of CKD following the initial repair, given that their numbers have been shown to remain elevated for up to 6 weeks following renal injury. In addition, we have recently shown that Th17 cells are markedly increased between weeks 5 and 9 post‐IRI in rats placed on a high‐salt diet to enhance hypertension and renal fibrosis (Mehrotra et al. 2015). Therefore, T cells may participate in long‐term structural changes in postischemic kidneys, thus promoting the transition from AKI to CKD (Ascon et al. 2009). In contrast, in other rodent IRI models, T‐regulatory cells have been shown to have positive effects on recovery by promoting tubular cell proliferation as well as exerting an antiinflammatory effect (Gandolfo et al. 2009). In this study, we found that the numbers of activated CD4^+^, CD8^+^, and Th17 cells after 7 days of recovery were dramatically higher in the VDD+IRI group than in the IRI rats on a standard diet, whereas VDD+IRI rats showed a reduced response to T‐regulatory cells. These results are consistent with the established data showing that vitamin D influences the immune system and that vitamin D deficiency results in increased activation of inflammatory cells.

In summary, vitamin D deficiency exacerbates vascular impairment and enhances inflammation during recovery from AKI. Whether the effects of vitamin D deficiency on these events following injury represent separate or interrelated activities is an important question to be addressed in future studies in order to gain a more complete understanding of the role of vitamin D in the transition to CKD.

## Conflict of Interest

None declared.

## Supporting information




**Figure S1.** Negative controls for S100A4/FSP‐1 (A and B), *α*‐SMA (C and D), and VEGF (E and F).
