# Supplementary figures and images for: Vitamin D deficiency contributes to vascular damage in sustained ischemic acute kidney injury
Source: Physiol Rep. 2016 Jul 1;4(13):e12829. doi: 10.14814/phy2.12829 (PMC4945834; doi:10.14814/phy2.12829)

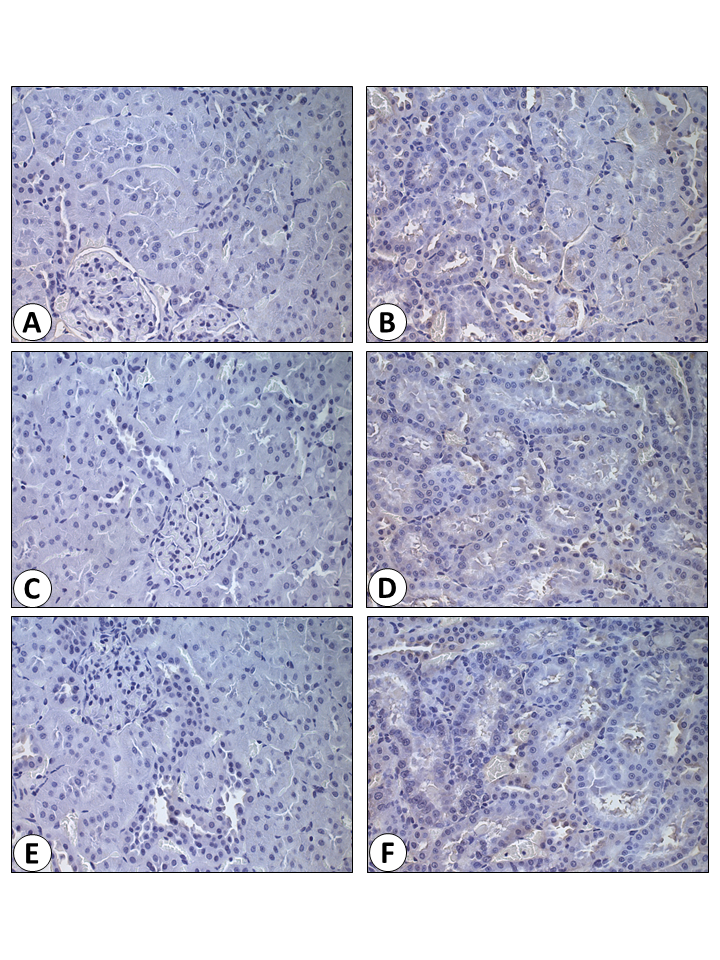

Supplement: Supplementary file 1 — Figure S1. Negative controls for S100A4/FSP‐1 (A and B), α‐SMA (C and D), and VEGF (E and F). [file PHY2-4-e12829-s001.tif]
